# Efficient and stable reconstitution of the ABC transporter BmrA for solid-state NMR studies

**DOI:** 10.3389/fmolb.2014.00005

**Published:** 2014-06-12

**Authors:** Britta Kunert, Carole Gardiennet, Denis Lacabanne, Daniel Calles-Garcia, Pierre Falson, Jean-Michel Jault, Beat H. Meier, François Penin, Anja Böckmann

**Affiliations:** ^1^Labex Ecofect, Bases Moleculaires et Structurales des Systemes Infectieux, UMR 5086 CNRS, IBCP, Université de Lyon 1Lyon, France; ^2^Physical Chemistry, ETH ZürichZurich, Switzerland

**Keywords:** solid-state NMR, ABC-transporter, BmrA, isotope labeling, *B. subtilis* lipids

## Abstract

We present solid-state NMR sample preparation and first 2D spectra of the *Bacillus subtilis* ATP-binding cassette (ABC) transporter BmrA, a membrane protein involved in multidrug resistance. The homodimeric 130-kDa protein is a challenge for structural characterization due to its membrane-bound nature, size, inherent flexibility and insolubility. We show that reconstitution of this protein in lipids from *Bacillus subtilis* at a lipid-protein ratio of 0.5 w/w allows for optimal protein insertion in lipid membranes with respect to two central NMR requirements, high signal-to-noise in the spectra and sample stability over a time period of months. The obtained spectra point to a well-folded protein and a highly homogenous preparation, as witnessed by the narrow resonance lines and the signal dispersion typical for the expected secondary structure distribution of BmrA. This opens the way for studies of the different conformational states of the transporter in the export cycle, as well as on interactions with substrates, via chemical-shift fingerprints and sequential resonance assignments.

## Introduction

Studies of membrane proteins in a membrane environment are key to understand their function at the molecular level, and solid-state NMR presents a promising method to address central structural questions. This emerging technique for the investigation of conformational details of insoluble and non-crystalline molecular assemblies has gone through major advances in the last years, and is on the way to become an important tool in structural biology (Böckmann, 2008; Lesage et al., 2008; Lorieau et al., 2008; Loquet et al., 2009; Böckmann and Meier, 2010; Jehle et al., 2010; Schuetz et al., 2010; Su et al., 2010; Van Melckebeke et al., 2010; Linser et al., 2011; Cukkemane et al., 2012). In principle, solid-state NMR is able to investigate large molecular assemblies, and does not require the presence of crystalline long-range order in three or two dimensions, nor soluble samples. It can investigate various kinds of biological material, as protein fibrils (Heise et al., 2005; Ferguson et al., 2006; Wasmer et al., 2008, 2009; Loquet et al., 2009; Barbet-Massin et al., 2010; Böckmann and Meier, 2010; Debelouchina et al., 2010; Helmus et al., 2010; Van Melckebeke et al., 2010; Comellas et al., 2011; Tycko, 2011; Sinnige et al., 2014), membrane proteins in different environments, including phospholipid bilayers (Andronesi et al., 2005; Lorch et al., 2005; Lange et al., 2006; McDermott, 2009; Shi et al., 2011; Emami et al., 2013), and 2D or 3D crystalline assemblies, regardless of the crystal size and diffraction properties. The initial studies using solid-state NMR techniques to characterize membrane proteins are very promising (Hiller et al., 2005; van Gammeren et al., 2005; Lange et al., 2006, 2010; Etzkorn et al., 2008; Li et al., 2008; Higman et al., 2009; Shi et al., 2009; Bhate et al., 2010; Cady et al., 2010).

The NMR methods mentioned above are based on the collection of distance and torsion-angle restraints which are then the basis of a structure calculation in analogy to the classical structure determination in solution-state NMR (Wüthrich, 2003). It is worth mentioning that for membrane proteins, solid-state NMR structure determination can alternatively be performed on the basis of orientational restraints obtained from measuring bond directions (e.g., for backbone amide N-H bonds) either in oriented samples (Ketchem et al., 1997; Opella et al., 1999; Marassi and Opella, 2000; Opella and Marassi, 2004; Claramunt et al., 2010; Sharma et al., 2010; Das et al., 2013; Cross et al., 2014; Murray et al., 2014a,b) or by rotationally oriented methods (Park et al., 2012; Das et al., 2014) which can also be combined with distance restraints.

Solid-state NMR is, by its capability of investigating proteins in their near-native environment and at physiological temperatures, complementary to other biophysical methods, as X-ray crystallography, which is currently the most successful method for protein 3D structure determination in general, and of ABC transporters in particular, and which will further benefit by the availability of free electron lasers (Barends et al., 2014). Importantly, recent considerable progress toward quasi-atomic resolution structures has also been made by cryo-electron microscopy (Liao et al., 2013; Kühlbrandt, 2014). NMR studies shall be able to contribute, in combination with other techniques, to obtain the most complete view on structures, as well as of dynamic behavior, of the important class of membrane proteins.

As is true for the other structural approaches as well, membrane protein sample preparation for solid-state NMR remains a challenge, which is in the context of solid-state NMR most noticeably due to the difficulties of membrane reconstitution, a central and critical step in sample preparation. To obtain spectra displaying narrow resonance lines, it is essential that all proteins are reconstituted in the membrane in the same manner, displaying a conformationally homogenous ensemble. Achieving membrane insertion with minimal protein loss is vital to maximize the NMR signal. Equally important, achieving long-term stability of reconstituted membrane proteins is fundamental to acquire highly resolved NMR spectra and to potentially repeat and optimize spectra over a period of several months.

ABC transporters are ubiquitous integral membrane proteins that energize the translocation of substrates across biological membranes by ATP hydrolysis, and are conserved from bacteria to humans (Higgins, 1992). These proteins work either as importers or exporters and are composed of two transmembrane domains (TMDs), forming the translocation pathway, and two highly conserved nucleotide-binding domains (NBDs), generating the energy for substrate translocation (Holland and Blight, 1999). Importers are almost only found in prokaryotes and require an extra-cytoplasmic binding protein for substrate recognition. In contrast, exporters are present in all organisms and can accept substrates directly from the cytoplasm or the lipid bilayer (Hollenstein et al., 2007). While some exporters are dedicated to transport a specific substrate (e.g., lipid A for MsbA Zhou et al., 1998), others remove a broad range of molecules from the cell, including xenobiotics such as antiviral, antibiotic, antifungal and anticancer agents in humans or antibiotics, antifungal or antiparasitic drugs in microorganisms. These multidrug transporters have thus been described as molecular “hydrophobic vacuum cleaners” and are promising therapeutic targets as they are involved in multidrug resistance phenotype (Ward et al., 2007; Aller et al., 2009). To find ways to modulate their activity in order to increase drug efficiency, it is necessary to understand how the transporters recognize such a wide variety of substrates (Martinez et al., 2014) and translocate them across the membrane during the catalytic cycle (Jones et al., 2009; Newstead et al., 2009; Ernst et al., 2010). Experience has shown that ABC exporters are extremely dynamic molecules (Hellmich et al., 2012; Mehmood et al., 2012), and the large conformational variability (Dawson and Locher, 2006, 2007; Ward et al., 2007; Aller et al., 2009; Hohl et al., 2012) including open, closed and several intermediate states makes a detailed structural characterization central to understanding their function.

The study of model transporter proteins of bacterial origin, for which overproduction yields quantities large enough for structural studies (i.e., several milligrams) and for stable isotopes enrichment (e.g., ^15^N, ^13^C), constitutes an important tool to further understand the building principles of their eukaryotic counterparts. First solid-state studies have been performed already a decade ago (Mason et al., 2004; Agarwal et al., 2007; Lehner et al., 2008) and a first high-resolution MAS solid-state NMR study of a thermophilic ABC importer, ArtMP from *Geobacillus stearothermophilus*, prepared in 2D crystalline form, has been reported recently (Lange et al., 2010). The authors have shown that amino-acid selective labeled ArtMP could be reconstituted in native lipid extracts yielding a homogenous solid-state NMR sample that gave rise to high-quality NMR spectra. Investigations, both in oriented media and by MAS NMR, of the homo-dimeric multidrug antiporter EmrE have also appeared recently (Gayen et al., 2013; Ong et al., 2013; Becker-Baldus and Glaubitz, 2014).

We here use the bacterial ABC exporter BmrA from *Bacillus subtilis* as a model. BmrA has been identified in the *B. subtilis* genome by homology search, displaying about 54% of identity, plus strong similarity with each half of the human P-glycoprotein (Steinfels et al., 2002), and 57% of identity plus strong similarity with the Sav1866 homodimer (Dawson and Locher, 2006). BmrA functions as a homodimer of ~130 kDa (Dalmas et al., 2005), and its amino-acid sequence is shown in Figure S1, together with secondary structure elements and functional sequences, predicted by homology from *E. coli* MsbA in its apo-form (Ward et al., 2007; Do Cao et al., 2009). BmrA, as other exporters, is constituted of four domains: 2 transmembrane domains with 6 predicted α-helices each, and two nucleotide binding domains (Dawson and Locher, 2006; Ward et al., 2007; Aller et al., 2009). We report here the first steps toward conformational studies of BmrA by solid-state NMR, including protein production and efficient reconstitution in lipids, as well as sample-quality assessment.

## Materials and methods

The detergents n-Dodecyl-β-D-maltopyranoside (DDM) and Triton X-100 were purchased from Anatrace (USA). The Ethylenediaminetetraacetic acid (EDTA)-free protease inhibitor cocktail came from Roche (Switzerland). PD10 columns and the Sephadex 200 10/300 GL were from GE Healthcare Life Sciences (UK) and Ni-NTA Agarose was from Qiagen (Germany). U-^13^C-D-glucose and ^15^N-ammonium chloride were purchased from Cortecnet (France) and Cambridge Isotope Laboratories, Inc. (USA). Phosphatidylcholine (EPC) and phosphatidic acid (EPA) from egg yolk came from Sigma Aldrich (USA). *E. coli* C41(DE3) competent cells were purchased from Lucigen (USA).

### Production and purification of BmrA

*BmrA* was expressed from the previously described pET23b(+)-*bmrA* plasmid (Steinfels et al., 2002) in the *E. coli* strain C41(DE3) (Miroux and Walker, 1996). The resulting protein carries a hexa-histidine affinity tag at its C-terminus and it contains three additional amino acid residues in the N-terminus coming from the cloning site (Figure S1). For the preparation of ^13^C, ^15^N labeled BmrA, bacteria were grown in M9-type medium (Studier, 2005). Isotope labeling was performed using uniformly 99% ^13^C-enriched glucose (2 g/L) and 98% ^15^N-enriched ammonium chloride (2 g/L) as sole carbon and nitrogen sources. A 50 mL ^13^C, ^15^N-M9-culture was inoculated with a 3 mL LB overnight culture that was started from a freshly transformed colony. This pre-culture was incubated at 37°C to a maximum optical density at 600 nm (OD_600_ nm) of 1.5 and was used to inoculate 150 ml ^13^C, ^15^N-M9 medium to an OD_600_ nm of 0.2, which was cultured at 25°C overnight. The two ^13^C, ^15^N-M9 pre-cultures were included to slowly adapt the bacteria to the final cultivation conditions. Afterwards the third pre-culture is diluted in 850 mL fresh ^13^C, ^15^N-M9 medium and incubated at 25°C, and expression was induced with 1 mM IPTG at an OD_600nm_ between 0.5 and 0.7. Cells were harvested 5–6 h after induction, in the beginning of the stationary growth phase, by centrifugation at 6000 × g.

Membrane fractions were prepared as described (Chami et al., 2002), suspended in buffer A (50 mM Tris-HCl pH 8.0, 1 mM EDTA, 300 mM sucrose), frozen in liquid nitrogen, and finally stored at −80°C. The purification follows the protocol described (Steinfels et al., 2002) with a few modifications. The membranes were thawed and diluted at 2 mg/mL protein with the solubilization buffer B: 50 mM Tris-HCl pH 8.0, 100 mM NaCl, 1% DDM, 1 mM Dithiothreitol (DTT), 15% glycerol, 5 mM MgCl_2_, Benzonase and EDTA-free protease inhibitor cocktail. The suspension was incubated for 1 h at 4°C and the insoluble material was removed by centrifugation at 98,000 ×g for 1 h at 4°C.

**Figure 1:**
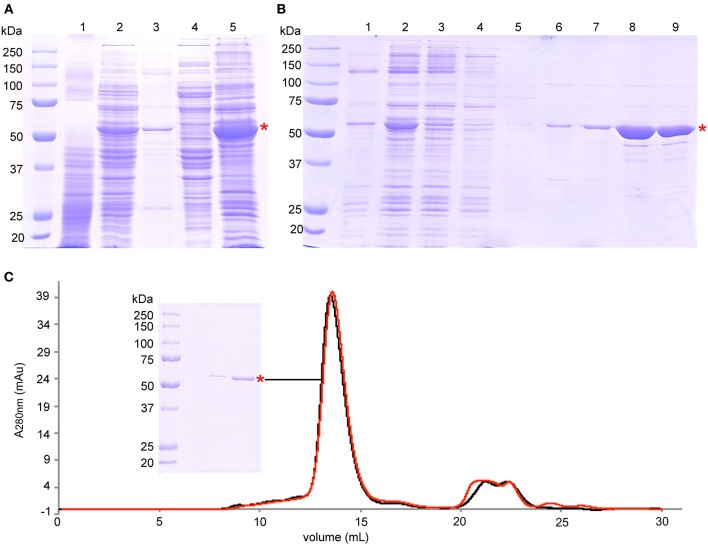
**Purification of BmrA. (A)** SDS-PAGE analysis of protein expression and membrane preparation: lane 1—cell lysate before induction, lane 2—cell lysate after 6 h of overexpression, lane 3—pellet after centrifugation at 15,000 × g, lane 4—supernatant after centrifugation at 193,000 × g, lane 5—isolated membranes after centrifugation at 193,000 × g. **(B)** SDS-PAGE analysis of protein solubilization and purification steps on Ni-NTA Agarose: lane 1—pellet after solubilization and centrifugation at 98,000 × g, lane 2—supernatant after solubilization and after centrifugation at 98,000 × g that was loaded onto Ni-NTA Agarose, lane 3—flow through of Ni-NTA Agarose gravity column, lane 4—wash step I with buffer C (0.5 mM NaCl, 10 mM imidazole), lane 5—wash step II with buffer C (0.1 M NaCl, 10 mM imidazole), lane 6—wash step III with buffer C (0.1 M NaCl, 30 mM imidazole), lane 7—wash step IV with buffer C (0.1 M NaCl, 40 mM imidazole), lane 8—elution with buffer C (0.1 M NaCl, 250 mM imidazole), lane 9—purified protein after removal of imidazole by PD10 columns. **(C)** Size exclusion chromatography of the final product after purification (black trace), and after 7 days at room temperature (red trace). SDS-PAGE of the BmrA peak is also shown. The peaks at 21 mL to 26 mL correspond mainly to nucleotides with absorption maxima of ~260 nm. BmrA is indicated by a red star. The SDS gels (10% acrylamide) were stained with Coomassie brilliant blue.

The solubilization efficiency for BmrA was about 80–90% (Figure 1A). The solubilized protein was loaded onto a Ni-NTA Agarose gravity column, which was equilibrated with buffer C (50 mM Tris-HCl pH 8.0, 100 mM NaCl, 15% glycerol, 0.2% DTT and 10 mM imidazole), washed successively with buffer C containing 0.5 M NaCl, with buffer C alone, buffer C containing 30 mM imidazole, buffer C containing 40 mM imidazole and finally eluted with buffer C containing 250 mM imidazole. The additional purification step with 0.5 M NaCl was introduced to reduce nucleotide contaminations. The buffer of the eluted protein was exchanged to buffer D (50 mM Tris-HCl, 100 mM NaCl, 10% glycerol, 0.2% DDM, EDTA-free protease inhibitor cocktail) using PD10 desalting columns (Figure 1B). The protein concentration was maintained in all steps at 2–3 mg/mL to avoid protein aggregation.

For subsequent reconstitution into lipids, BmrA was diluted to a final concentration of 0.2 mg/mL and 0.05% DDM. Protein concentration was determined by absorbance at 280 nm. The final preparation was analyzed by analytical size exclusion chromatography (SEC) using a Sephadex 200 10/300 GL column (Figure 1C). SEC was performed using buffer D containing 0.05% DDM at a flow rate of 0.3 mL/min. All purification steps were followed by Coomassie brilliant blue-stained 10% sodium dodecyl sulfate polyacrylamide gel electrophoresis (SDS-PAGE) (Figure 1). The final yield of BmrA was around 10 mg per liter of culture. The main band on SDS-PAGE was digested by trypsin and analyzed by LC-MS/MS to ensure that it contains exclusively BmrA.

### Lipid extraction from *B. subtilis*

*B. subtilis* was cultivated in LB medium (Sigma Aldrich) at 37°C for 18 h. The cells were harvested at an OD_600nm_ of 4 by centrifugation at 6000 × g. Lipid extraction was done as described (Bligh and Dyer, 1959). In short, 10 g bacterial pellets were suspended in 60 ml chloroform and methanol (1:2, v/v). The mixture was stirred for 2 h at room temperature. Solid particles were removed by filtration. The filtrate was mixed with 20 mL chloroform and 20 mL deionized water and extraction was performed for 2 days in a separation funnel. The solvent of the lower phase was removed in a rotary evaporator and by lyophilization. The lipids were stored in chloroform at −20°C for no longer than 1 month. The final lipid amount was about 20 mg per liter of *B. subtilis* culture.

### Reconstitution of BmrA into lipids from *B. subtilis* and into EPC/EPA

Lipids were solubilized in chloroform and Triton X-100 in methanol. Then, lipid/chloroform and Triton X-100/methanol were mixed in a lipid-detergent ratio 1:10 (mol/mol). An average molecular weight of 768 Da was taken as a basis for the calculations for the mixture of egg-L-α-phosphatidylcholine (EPC)/ egg-L-α-phosphatidic acid (EPA), and ~1000 Da for the *B. subtilis* lipid extracts (Griffiths and Setlow, 2009). EPC and EPA were used in a ratio 9:1 (w/w) as described (Orelle et al., 2008). Natural lipids were preferred over synthetic lipids, as they display a range of fatty acid chain lengths and degrees of saturation (Avanti Polar Lipids Product Sheet).

The solvents were removed by N_2_ gas flow, followed by lyophilization for 30 min. The resulting lipid/detergent film was first dissolved in ½ final volume deionized H_2_O and afterwards in ½ final volume buffer E (100 mM Tris-HCl pH 8.0, 200 mM NaCl, 20% glycerol) to a final lipid concentration of 5 mg/mL. The lipids were added to the detergent solubilized BmrA (0.2 mg/mL) to a LPR (lipid-to-protein ratio) of 0.5, 1, or 2. Subsequently, the detergents were removed by dialysis with buffer F (50 mM Tris-HCl pH 8.0, 200 mM NaCl, 5% glycerol, 0.05% NaN_3_) for 9 days at room temperature. 40 mL of sample were dialyzed in 5 L of buffer J using dialysis tubing with a cut-off of 6–8 kDa (Spectra/Por^®^ Dialysis Membrane, SPECTRUMLABS.COM). To ensure an efficient depletion of detergents, three times the theoretical required quantity of Bio-Beads (SM2, 20–50 mesh; Bio-Rad, USA) was added to the dialysis buffer at the beginning of dialysis and again after 6 days. The calculations were based on the adsorption capacities of 105 mg DDM per 1 g Bio-beads (Rigaud et al., 1995) and 70 mg Triton X-100 per 1 g Bio-Beads (Bio-Rad, user manual). Incorporation of BmrA into lipid membranes was analyzed by centrifugation on sucrose gradient (see below), transmission electron microscopy (TEM) and coomassie brilliant blue stained SDS-PAGE (10% acrylamide). For analysis by SDS-PAGE, a 100 μl sample was centrifuged at 20,000 × g, 4°C for 30 min. The pellet was resuspended in 100 μl of buffer F. The TEM analysis was done by the Center d'Imagerie Quantitative Lyon Est (CIQLE, France) on a TEM JOEL 1400. Samples were negatively stained with 2% phosphotungstic acid. The protein concentration was determined with BCA Protein Assay Kit from Pierce (USA). A 3.2 mm NMR rotor (Bruker Biospin) was filled with reconstituted BmrA by centrifugation at 30,000 × g using a custom-made device (Böckmann et al., 2009).

### Analysis of reconstituted BmrA in a sucrose gradient

A discontinuous sucrose density gradient was prepared by layering 200 μL of 10, 20, 25, 30, 35, 40, 45, 50, 60, and 70% (w/v) sucrose in 50 mM Tris-HCl pH 8.0, 200 mM NaCl, 5% glycerol. A sample volume of 400 μL was layered on top of the gradient and centrifuged at 164,000 × g at the average radius of 91.7 mm in a Beckman SW 60 Ti rotor during 16 h at 4°C. After centrifugation, the different fractions were collected by pipetting and analyzed by SDS-PAGE (10% acrylamide).

### ATPase activity assay

For ATPase activity measurements, 4–6 μg reconstituted BmrA in 25 μl buffer F were pre-incubated at 37°C for 1 min before 10 mM ATP/10 mM MgCl_2_ were added. 0.8 mM ortho-vanadate (Sigma Aldrich) was added 1 min after activation with ATP/MgCl_2_ to inhibit BmrA (Liu et al., 1997). After 4 min, the reaction was stopped by addition of an equal amount of 12% sodium dodecyl sulfate and the ATP hydrolysis was determined by measuring free Pi (Chifflet et al., 1988).

### Solid-state MAS NMR experiments

All NMR experiments were carried out on a Bruker Biospin AVANCE III spectrometer operating at 800 MHz ^1^H frequency using a 3.2 mm triple-resonance (^1^H, ^13^C, ^15^N) E-free probe (Bruker Biospin). Sample temperature was determined using the chemical shift of supernatant water (Böckmann et al., 2009) and was set to 278 K. All experiments were conducted at 17.5 kHz spinning frequency using 90 kHz SPINAL64 (Fung et al., 2000) proton decoupling. The proton and carbon field amplitudes during the ^1^H-^13^C cross-polarization transfer step were around 66 kHz and 50 kHz respectively, using a tangential ramp with a span of ±5% on the carbon channel. The Dipolar Assisted Rotational Resonance (DARR) (Takegoshi et al., 2001; Morcombe et al., 2005; Scholz et al., 2008) experiment was recorded in 48 scans per increment with a 20 ms mixing time. Acquisition times were 15 ms in t_2_ and 10 ms in t_1_, and the interscan delay was 2 s, corresponding to a total measurement time of 50 h. Spectra were processed with Topspin using a shifted cos^2^ function and analyzed using CcpNmr Analysis (Stevens et al., 2011).

## Results and discussion

### BmrA is produced in high yield and high purity

For production of isotopically labeled BmrA required for NMR studies, we adapted the protocol (Steinfels et al., 2002) for overexpression of the protein in minimal medium. Under these conditions, the yield for BmrA was 10 mg of purified protein per liter of culture. The results of protein production, membrane preparation and purification, analyzed by SDS-PAGE, are shown in Figure 1. BmrA could be solubilized efficiently from the *E. coli* membranes by DDM (up to 80%). Protein loss was successfully avoided by ensuring that the high transient protein concentrations during purification were accommodated by an increased detergent concentration during affinity chromatography. It was however important that, before reconstitution into lipid membranes, the detergent concentration was reduced, to avoid even longer dialysis times. The final product did not contain BmrA aggregates and was stable at room temperature over at least 7 days, as shown by analytical size exclusion chromatography (Figure 1C). The main band on SDS-PAGE is exclusively composed of BmrA from *B. subtilis* as confirmed by mass spectrometry analysis.

### Slow detergent removal by dialysis yields homogenous BmrA samples

**Figure 2:**
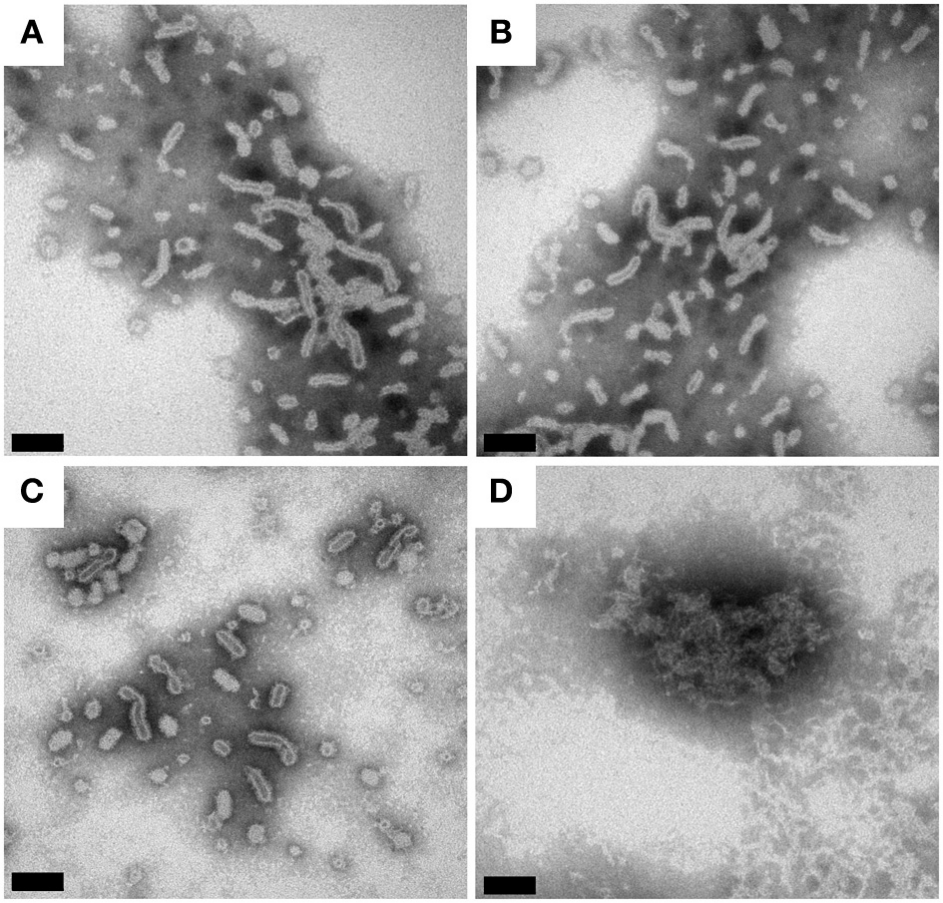
**Transmission electron micrographs of BmrA in *B. subtilis* lipids and EPC/EPA (9:1)**. The protein reconstituted into a lipid bilayer forms ring- and tubular structures (Orelle et al., 2008). **(A)** BmrA in *B. subtilis* lipids 1 day after dialysis. **(B)** BmrA in *B. subtilis* lipids 15 days after dialysis (stored at room temperature). **(C)** BmrA in EPC/EPA 1 day after dialysis. **(D)** BmrA in EPC/EPA 15 days after dialysis (stored at room temperature), showing the loss of the tubular structure and protein aggregation indicating that, in contrast to the sample in native lipids, the sample is not stable in EPC/EPA for long-term storage. The samples were negatively stained with 2% phosphotungstic acid. The bars represent 100 nm **(A–C)** and 50 nm **(D)**.

BmrA can be inserted in lipid membranes of different composition (EPC/EPA, DMPC, DOPC and E. coli lipids) and form ring-shaped or tubular superstructures (Orelle et al., 2008; Dezi et al., 2011; Galián et al., 2011; Fribourg et al., 2014). A recent 3D cryo-electron reconstruction of BmrA in this ring-shaped structures clearly shows that the homodimer is embedded in a lipid bilayer. The NBDs are located on the outside of the ring. The 3D model of the apo-BmrA shows an open inward-facing conformation (Fribourg et al., 2014). The formation of rings vs. tubes depends on the lipid-to-detergent ratio in the sample. On further detergent removal, the rings tend to stack together forming non-crystalline tubular structures (Chami et al., 2002; Fribourg et al., 2014). As BmrA is a protein from *B. subtilis*, we tried *B. subtilis* lipid extract to prepare BmrA in a lipid environment as close as possible to the native conditions. The cytoplasmic membrane of *B. subtilis* is mainly composed of phosphatidylglycerol (23%), cardiolipin (46%), phosphatidylethanolamine (14%) and diglucosyl diacylglycerol (17%) (Griffiths and Setlow, 2009). An EPC/EPA mixture at 9:1 (w/w) was used for comparison, as it has already been successfully used for cryo-EM studies (Chami et al., 2002; Fribourg et al., 2014). For reconstitution of BmrA in lipids, the detergent-solubilized protein is mixed with the detergent-solubilized lipids (Rigaud et al., 1995). While in the previously published protocol [used for the preparation of electron-microscopy (EM) samples], the detergent removal was done by subsequent addition of Bio-Beads directly to the protein-lipid-detergent mixture (Orelle et al., 2008), the use of this protocol resulted in about 50% loss of protein using a LPR of 0.5. This is likely due to a sudden and inhomogeneous removal of detergents by Bio-Beads, leading to protein aggregation. We thus used dialysis for detergent removal, with Bio-Beads added in the dialysis buffer outside the dialyzed sample in order to deplete the detergent. This procedure yielded homogenous samples displayed in the EM micrographs in Figures 2A,C. The samples contained mainly non-crystalline supra-molecular tubular structures with *B. subtilis* lipids as well as with EPC/EPA, consistent with the previous studies (Orelle et al., 2008; Dezi et al., 2011; Galián et al., 2011; Fribourg et al., 2014). 2D crystals of BmrA have been described in the literature only for the protein in the ADP-Vi trapped state (Fribourg et al., 2014).

### BmrA is stable when reconstituted in native *B. subtilis* lipids

**Figure 3:**
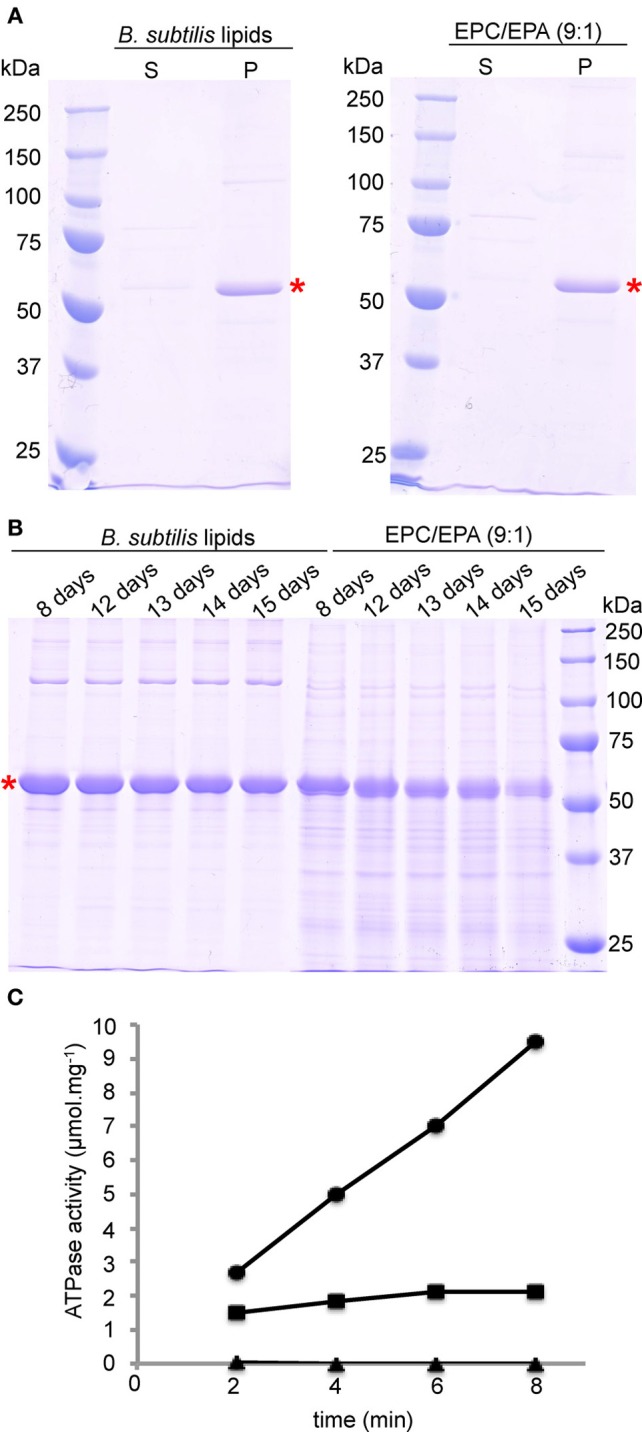
**Reconstitution and stability of BmrA in *B. subtilis* lipids and EPC/EPA (9:1) analyzed with SDS-PAGE. (A)** After 9 days of dialysis, an aliquot was centrifuged at 20000 × g for the analysis of the supernatant (S) and the pellet (P). **(B)** For analysis of the stability of BmrA in the different lipid environments, after dialysis protein-lipid structures were incubated at room temperature and aliquots were analyzed by SDS-PAGE at the indicated times. BmrA is indicated by a red star. The SDS-PAGE gels (10% acrylamide) were stained with Coomassie brilliant blue. **(C)** Typical results for ATP hydrolysis by BmrA (dots) and its efficient inhibition by ortho-vanadate (rectangles). As a control BmrA was measured without additives (triangles). Samples were analyzed after 2, 4, 6, and 8 min after addition of ATP/MgCl_2_.

The SDS-PAGE gels of reconstituted BmrA in *B. subtilis* lipids and in EPC/EPA lipids are shown in Figure 3A. After 9 days of membrane reconstitution, the protein is mainly observed in the pellet, for both lipid mixtures. Figure 3B shows the evolution of the sample with time, an important parameter for solid-state NMR samples, which are often used over long time periods. One can see that the preparation in *B. subtilis* lipids, when kept at room temperature, is stable over 2 weeks and free of degradation. In EPC/EPA (9:1), the intensity of the BmrA band decreases over time, indicating a slow degradation. An analysis by EM of the evolution of the samples in *B. subtilis* lipids (Figures 2A, B) and EPC/EPA (9:1) (Figures 2C,D) confirms that the preparation in the native lipids is stable over 2 weeks at room temperature, while the EM after 15 days in EPC/EPA (9:1) shows the loss of tubular structures and the appearance of aggregated protein (Figure 2D). The higher stability of tubes containing BmrA in *B. subtilis* lipids might arise from their lipid composition. The same protein stability would possibly be achieved as well using more complex lipid mixtures from natural sources or synthetic lipids with a similar composition.

Importantly, the NMR samples prepared using *B. subtilis* lipids are stable over several months (see below). As a further criterion for sample quality we routinely address the ATPase activity of the preparations. The presented protocol for reconstitution of BmrA in *B. subtilis* lipids with a LPR of 0.5 gives here a highly reproducible ATPase activity of 1.3 ± 0.1 μmol hydrolyzed ATP per minute and mg BmrA, that can be inhibited by ortho-vanadate to 95 ± 8% (Figure 3C). These results are comparable to previously published data for ring and tube like structures of BmrA in lipid membranes (Orelle et al., 2008).

### A lipid-to-protein ratio of 0.5 is optimal

**Figure 4:**
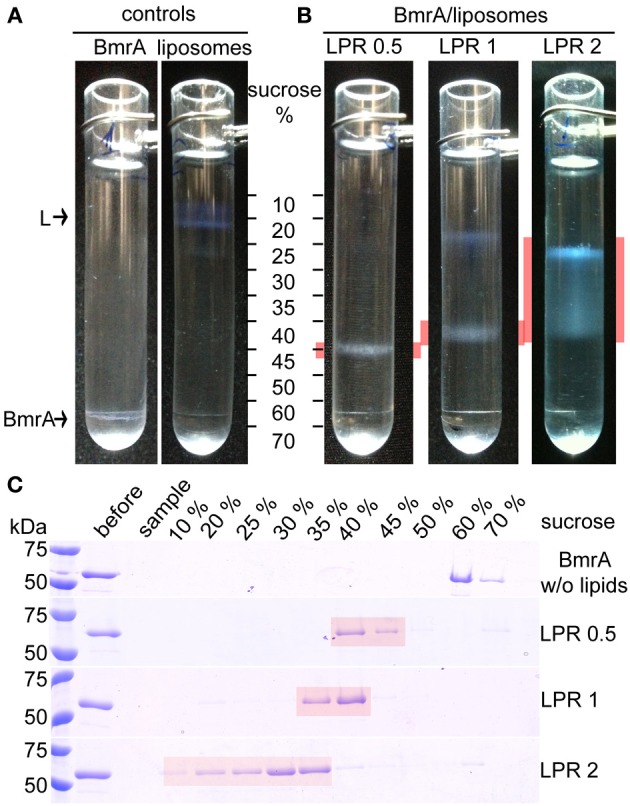
**Analysis of BmrA reconstitution in *B. subtilis* lipids with different LPRs by sucrose gradient centrifugation. (A)** Controls: Sucrose gradients of BmrA dialyzed in the absence of lipids in order to identify the densities of aggregated BmrA (left) and of lipids without protein (right, labeled by an “L”). **(B)** Sucrose gradients of BmrA reconstituted in *B. subtilis* lipids using LPR 0.5, 1 and 2. Gradient layers containing BmrA in lipids are highlighted in red. **(C)** The layers (~200 μl) of the gradients were analyzed by SDS-PAGE. The first two lanes after the protein ladder show the sample before centrifugation (before) and the sample-layer (sample) after centrifugation. Given concentrations correspond to sucrose concentrations before centrifugation.

Measuring the protein concentration before and after reconstitution reveals that when a LPR of 0.5 is used, about 90% of the protein is reconstituted into lipids, both when *B. subtilis* lipids or EPC/EPA were used. This is also shown in the SDS-PAGE gel in Figure 3A, where the majority of the protein is observed in the pellet. Higher LPRs were tested to reduce protein loss and obtain complete insertion into lipid membranes, but such conditions resulted in NMR samples with less signal/noise. Besides the increased amount of lipids, the loss also resulted from an increased sample viscosity, which yielded on rotor packing a gluey, very hydrated pellet. Using a sucrose density gradient, we visualized the fractions containing only lipids (in absence of protein), tube-like protein-lipid structures and protein aggregates, as these species show a different sedimentation behavior. Figure 4 shows the sucrose density gradient analysis, comparing BmrA reconstituted in *B. subtilis* lipids at LPR of 0.5, 1 and 2. The first centrifugation tube (Figure 4A) shows a BmrA-without-lipids control, which aggregates upon detergent removal and is found between 60 and 70% sucrose, as checked by SDS-PAGE (Figure 4C, top). The second centrifugation tube (Figure 4A) shows a lipids-without-protein control, found around 10–20% sucrose. In Figure 4B, lipid-inserted protein can be seen as a white band in-between these bands in the control experiment. At an LPR of 0.5, only lipid-protein structures are observed, with a narrow band and a homogenous density around 40–45% sucrose. Using a LPR of 1, one can clearly observe lipids containing almost no protein around 10–20% sucrose, and a broader band of supra-molecular structures around 35–40% sucrose, indicating a heterogeneous composition. By increasing the LPR to 2, a wider distribution of this band appears between 10 and 40% sucrose, with protein concentration increasing from top to bottom (Figure 4C, bottom).

### Optimized BmrA samples yield high-quality solid-state NMR spectra

**Figure 5:**
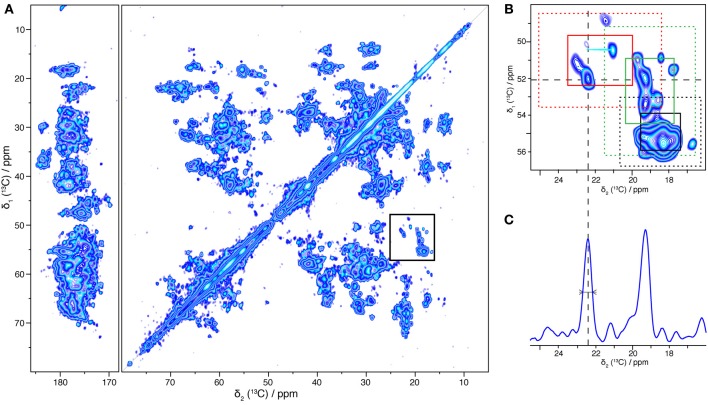
**^13^C-^13^C two-dimensional solid-state NMR correlation experiment (DARR, 20 ms). (A)** Overview spectrum with aliphatic and carbonyl regions. **(B)** Alanine region. Regions typical for β-sheet, α-helical and coil alanine signals are indicated respectively by red, black and green rectangles: solid lines correspond to the mean value +/− standard deviation and dashed lines to the mean value +/− 2 times the standard deviation (Wang and Jardetzky, 2002). **(C)** 1D trace taken at 52.1 ppm, the linewidth at half height is 0.5 ppm.

To summarize the optimal preparation conditions used for the NMR sample: the protein was reconstituted in *B. subtilis* lipids, starting from 0.2 mg/mL BmrA in 0.05% DDM, using a LPR of 0.5, and detergent removal was conducted by dialysis with Bio-Beads added to the buffer, for 9 days. A two-dimensional ^13^C-^13^C DARR correlation spectrum of the resulting BmrA sample is shown in Figure 5A. The spectrum displays narrow resonance lines for isolated peaks, and a chemical-shift dispersion typical for a well-folded protein, even if the large number of signals from the 589 amino-acid-residue protein results in heavy resonance overlap. Cross-peaks from many individual atom pairs can be detected. Isolated resonances typically exhibit a line width of 0.5 ppm, and a signal-to-noise ratio of 8.75, as shown for example for cross peaks stemming from Ala Cα-Cβ (Figure 5C). The signal-to-noise ratio in this 50 h experiment is excellent in the sense that the signal-to-noise per unit time of an isolated residue (the Ala peak highlighted in Figure 5C) is, taking into account the size of the protein but not yet the dilution by lipids, within a factor of 2 of the one of a microcrystalline ubiquitin sample, indicating a good rotor filling and a protein state that shows efficient cross polarization and DARR polarization transfer.

The chemical shift is sensitive to the secondary structure; it enables to differentiate Cα-Cβ cross-peaks originating from α-helices, β-sheets, and coils. The overall distribution, for the example of alanine chemical shifts, is shown in Figure 5B. Regions typical for alanines in α-helices, coils and β-sheets (Wang and Jardetzky, 2002) are indicated as red, green and black rectangles, respectively. The distribution suggests that BmrA shows a secondary structure organization similar to that described for MsbA in its apo form (PDB ID 3B5W). Indeed, a BmrA model built by homology from MsbA (Do Cao et al., 2009) predicts 30 alanine residues in α-helices (20 from the TMD and 10 from the NBD), 2 in β-sheet (from the NBD) and 13 in coils (4 from the TMD and 9 from the NBD). Figure 5B clearly shows, besides an intense α-helical cross signal, alanine residues in β-sheet and coil conformation, which should mostly stem from the nucleotide-binding domain. This clearly indicates that both the TMD and the NBD are visible in this spectrum.

**Figure 6:**
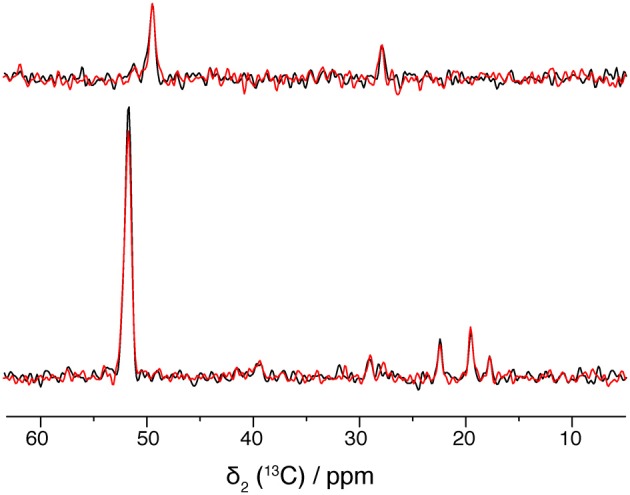
**1D traces at 49.5 ppm (top) and 51.8 ppm (bottom) of 20 ms DARR spectra from a freshly prepared BmrA apo sample (in black) and from a same sample after storage at 4°C for several months (in blue)**. Both spectra were recorded with 16 scans.

Solid-state NMR requires high sample stability, as spectra are often measured over several days or weeks at temperatures above 0°C. Figure 6 compares spectra of freshly prepared BmrA and a sample after storage at 4°C for several months. The 2D spectrum shows identical 2D ^13^C-^13^C correlations, as well as same line-widths, as shown in the 1D traces in Figure 6. It is worth mentioning as well that the sample preparation yields excellent reproducibility, as spectra from different protein/lipid preparations at different points in time display the same fingerprint.

## Conclusion

We report here NMR-scale expression and lipid reconstitution of the *B. subtilis* ABC transporter BmrA leading to a lipid-inserted sample of uniformly [^13^C, ^15^N] enriched BmrA. The preparation yields narrow resonance lines in the NMR-spectra rivaling microcrystalline preparations (McDermott et al., 2000). The excellent quality of the NMR spectra is a good indicator for a structurally homogeneous membrane-insertion of the protein. Our results demonstrate the feasibility of studying BmrA, and related membrane proteins, by solid-state NMR to obtain site-resolved information about structural and dynamic features of membrane transport proteins in different states (open, closed, drug-bound), even if the *de novo* structure determination of a protein of this size is still extremely challenging. BmrA is one of the largest proteins studied today by solid-state NMR, and has a typical size encountered for most membrane proteins. We are therefore very optimistic about the further prospects for solid-state NMR of membrane proteins in close-to-natural lipid environment.

## Conflict of interest statement

The authors declare that the research was conducted in the absence of any commercial or financial relationships that could be construed as a potential conflict of interest.
