# Supplementary material for: Efficient and stable reconstitution of the ABC transporter BmrA for solid-state NMR studies
Source: Front Mol Biosci. 2014 Jun 12;1:5. doi: 10.3389/fmolb.2014.00005 (PMC4428385; doi:10.3389/fmolb.2014.00005)
Supplement: Supplementary file 1 [file Presentation1.PDF]

Frontiers in Molecular Biosciences

Original Research
2014-04-07

Supplementary Material

Efficient and stable reconstitution of the ABC transporter BmrA for solid-state NMR studies

Britta Kunert[1#], Carole Gardiennet[1#], Denis Lacabanne[1], Daniel Calles-Garcia[1], Pierre Falson[1], Jean-Michel Jault[1], Beat H. Meier[2], François Penin[1] and Anja Böckmann[1*]

[1] IBCP, Bases Moléculaires et Structurales des Systèmes Infectieux, UMR 5086 CNRS/Université de Lyon 1, Labex Ecofect, 7 passage du Vercors, F-69367, France
[2] Physical Chemistry, ETH Zürich, Wolfgang-Pauli-Strasse 10, CH-8093 Zurich, Switzerland

*** Correspondence:** Anja Böckmann, IBCP, Bases Moléculaires et Structurales des Systèmes Infectieux, UMR 5086 CNRS/Université de Lyon 1, 7 passage du Vercors, F-69367, France. a.bockmann@ibcp.fr

1. Supplementary Figures

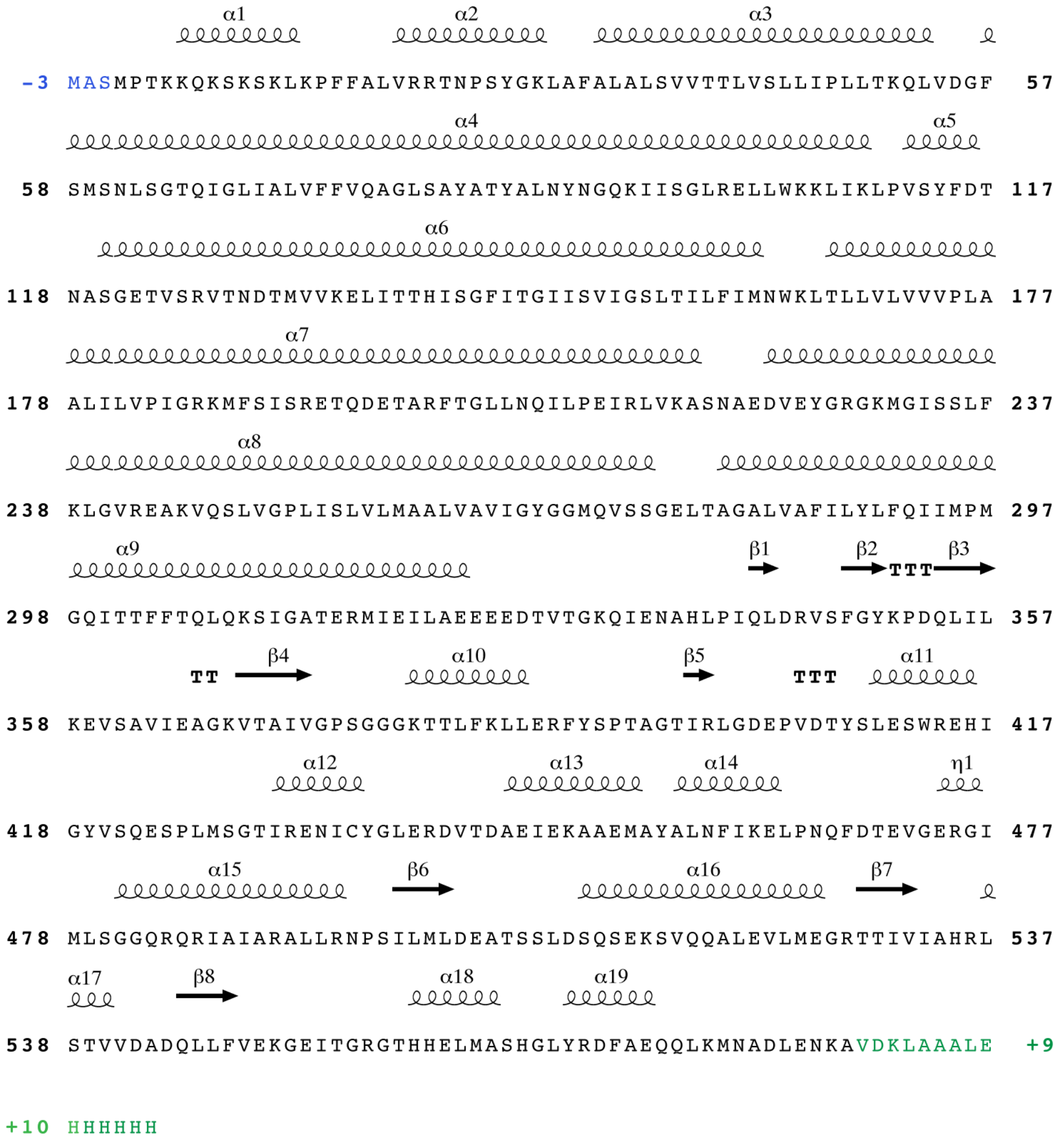

Figure S1: Amino acid sequence of the wild-type BmrA and secondary structure predictions. The transmembrane domain (grey) and the nucleotide-binding domain (black) are linked by a putative loop (red). The protein derived from pET23b(+)-BmrA carries 3 additional N-terminal (blue) and 15 C-terminal residues containing the hexa-histidine tag (green) (Steinfels et al., 2002). The secondary structure visualization was done by ESPript 3.0 (Gouet et al., 2003) based on a previously reported homology model on the apo-form of MsbA (Do Cao et al., 2009).

Do Cao, M.-A., Crouzy, S., Kim, M., Becchi, M., Cafiso, D. S., Di Pietro, A., and Jault, J.-M. (2009). Probing the

conformation of the resting state of a bacterial multidrug ABC transporter, BmrA, by a site-directed spin labeling approach. *Protein Sci.* 18, 1507–1520. doi:10.1002/pro.141.

Gouet, P., Robert, X., and Courcelle, E. (2003). ESPript/ENDscript: Extracting and rendering sequence and 3D information from atomic structures of proteins. *Nucleic Acids Res.* 31, 3320–3323.

Steinfels, E., Orelle, C., Dalmas, O., Penin, F., Miroux, B., Di Pietro, A., and Jault, J.-M. (2002). Highly efficient over-production in E. coli of YvcC, a multidrug-like ATP-binding cassette transporter from Bacillus subtilis. *Biochim. Biophys. Acta* 1565, 1–5.
